# LC–MS assay for quantifying ornithine decarboxylase activity in the biological matrix and cultured cells using stable isotope‑labeled ornithine

**DOI:** 10.1007/s00726-026-03539-9

**Published:** 2026-06-21

**Authors:** Yuma Shiomi, Naoya Ogawa, Kentaro Takahama, Atsushi Murai, Kyohei Furukawa

**Affiliations:** 1 Laboratory of Animal Nutrition, Department of Animal Science, Graduate School of Bioagricultural Science, Nagoya University, Furo-cho, Nagoya, 464-8601 Aichi Japan; 2 Technical Center, Nagoya University, Furo-cho, Nagoya, 464-8601 Aichi Japan

**Keywords:** Ornithine decarboxylase, Polyamines, Dansyl chloride, Stable isotope, LC–MS, DFMO

## Abstract

Polyamines (putrescine, spermidine, and spermine) are essential for animal health and development, and their intracellular levels must be tightly regulated to maintain normal cellular functions. Ornithine decarboxylase (ODC) catalyzes the rate-limiting decarboxylation step in polyamine biosynthesis, and thus accurate assessment of its activity is vital for studies of polyamine metabolism. However, conventional ODC assays rely on radiolabeled substrates and require specialized facilities for radioactive handling. To overcome these limitations, we developed a sensitive and non-radioactive ODC assay using stable isotope-labeled ornithine in combination with liquid chromatography–mass spectrometry (LC–MS). In this protocol, animal tissues (e.g., 0.5 g) or cultured cells (e.g., 2.0 × 10^6^ cells) are homogenized, and cytosolic fractions are prepared by centrifugation. These fractions are incubated with d_7_-ornithine under 37 °C conditions, and the produced d_7_-putrescine is derivatized with dansyl chloride and quantified by LC–MS analysis. Reaction samples showed a distinct peak corresponding to d_7_-putrescine, whereas negative control displayed negligible signals. Moreover, d_7_-spermidine and d_7_-spermine were not detectable under these conditions, indicating d_7_-putrescine production directly reflects ODC activity. We optimized reaction time and substrate concentrations to ensure linearity and precision, and confirmed that the assay responds appropriately to pharmacological inhibition of ODC. Collectively, this protocol provides a practical, sensitive, and non-radioactive method for quantifying ODC activity in both animal tissues and cultured cells, and it offers an accessible tool for polyamine metabolism research.

**Supplementary Information:**

The online version contains supplementary material available at https://doi.org/10.1007/s00726-026-03539-9.

## Introduction

Polyamines (putrescine, spermidine, and spermine) are ubiquitous polycationic molecules that are essential for animal growth, development, and health (Morgan 1999; Agostinelli 2020; Xuan et al. 2023; Hofer et al. 2024). Because of their strong positive charge at physiological pH, polyamines interact with polyanionic macromolecules such as DNA and RNA as well as proteins including kinases and ion channels (Forsythe 1995; Igarashi and Kashiwagi 2000; Leroy et al. 1997). Through these interactions, polyamines influence gene expression, post-transcriptional regulation, cell cycle progression, and membrane structure and function (Igarashi and Kashiwagi 2000, 2021). Polyamine levels decline with aging in mammals and reduced polyamine availability has been associated with age-related physiological abnormality (Hofer et al. 2024; Minetti et al. 2025; Minois et al. 2011). In contrast, cells with high proliferative capacity, such as cancer cells and microorganism, exhibit increased polyamine synthesis to support accelerated growth (Gerner and Meyskens 2004). Thus, appropriate regulation of polyamine metabolism is vital for both maintaining health and preventing abnormal cell growth.

**Fig. 1:**
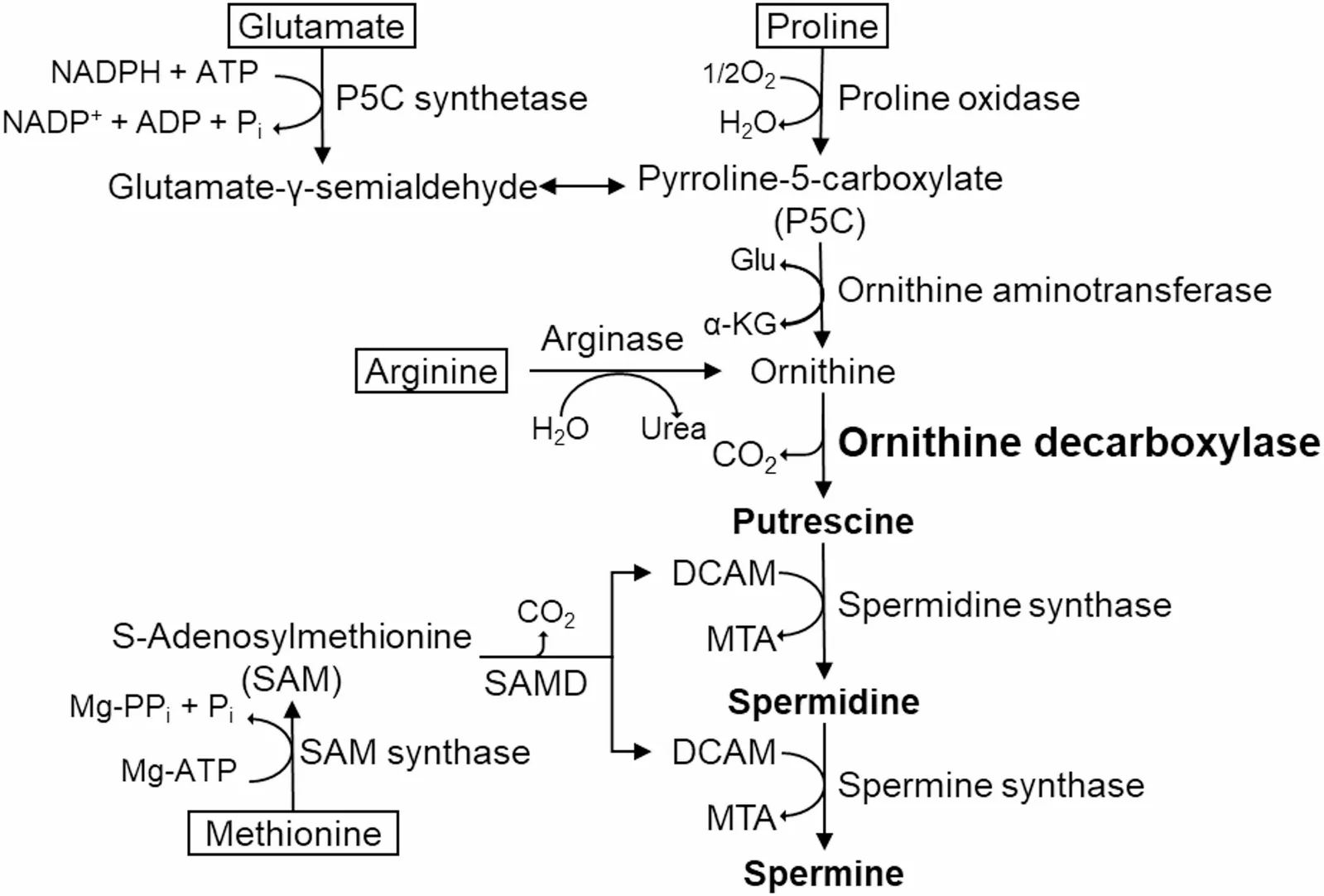
Endogenous polyamine synthesis pathway. Putrescine is synthesized from ornithine catalyzed by ornithine decarboxylase (ODC), and ornithine is produced from proline or arginine. Putrescine is subsequently converted to spermidine and spermine using decarboxylated S-adenosyl methionine (DCAM) derived from methionine. Glu: glutamate, α-KG: α-ketoglutaric acid, P5C: pyrroline-5-carboxylate, SAM synthase: S- adenosylmethionine synthase, SAMD: S-adenosylmethionine decarboxylase, DCAM: decarboxylated 5-adenosylmethionine, MTA: methylthioadenosine

Polyamines in animals originate from dietary intake, gut microbiota and endogenous synthesis (Muñoz-Esparza et al. 2019; Wu 2013). As shown in Fig. 1, endogenous polyamine synthesis involves amino acid metabolism that generates ornithine from arginine or from proline/glutamate intermediates such as pyrroline-5-carboxylate. Ornithine decarboxylase (ODC) then catalyzes the decarboxylation of ornithine, initiating the putrescine synthesis. Spermidine and spermine are subsequently synthesized through aminopropyl transfer reaction using S-adenosylmethionine-derived metabolites. Since ODC functions as the rate limiting enzyme of polyamine synthesis, its activity is tightly regulated at transcription, translation and protein degradation level (Pegg 2006; Wu et al. 2015; Snapir et al. 2008). ODC activity is also upregulated in highly proliferative cells and proliferative diseases (Wu et al. 2015; Snapir et al. 2008), making accurate evaluation of its activity essential for polyamine research and biomedical application.

Conventional assays for ODC activity predominantly rely on radiometric methods using [^14^C]- or [^3^H]- ornithine (Djurhuus 1981; Beeman and Rossomando 1989). In these assays, the released labeled CO₂ is trapped and quantified by scintillation counting. While these assays are highly sensitive, they require specialized infrastructure, radioactive handling procedures, and regulated waste disposal, which limits their utility in many laboratories. Therefore, practical and accurate non-radioactive alternatives are needed.

In the present study, we established a non-radioactive ODC activity assay using stable isotope-labeled ornithine combined with LC–MS detection. This method overcomes the safety and accessibility limitation of radiometric assays while maintaining high analytical specificity and sensitivity.

## Materials

### Reagents

Ultrapure water (H_2_O) (Fujifilm Wako; Cat. # 214–01301).

Methanol for LC/MS (Methanol) (Fujifilm Wako; Cat. # 138-14521).

1,4-butane-d_8_-diamine dihydrochloride (d_8_-putrescine) (Toronto Research Chemicals; Cat. # D416021).

Putrescine dihydrochloride (Sigma-Aldrich; Cat. # P7505-100 G).

Spermidine trihydrochloride (Sigma-Aldrich; Cat. # S2501-1 G).

Spermine tetrahydrochloride (Sigma-Aldrich; Cat. # 85605-1 G).

L-Ornithine-d_7_ hydrochloride (d_7_-ornithine; 99.9% isotopic purity) (MedChemExpress; Cat. # HY-W017018S4; seven deuterium atoms located at the α-carbon (one position) and β-, γ-, and δ-carbons (two positions each))

2-Amino-2-hydroxymethyl-1,3-propanediol (Tris) (Fujifilm Wako; Cat. # 201–06273).

Hydrochloric Acid (HCl) (Fujifilm Wako; Cat. # 080-01066).

Pyridoxal 5’-phosphate hydrate (Sigma-Aldrich; Cat. # P9255-1 G).

Eflornithine hydrochloride monohydrate (DFMO) (Thermo Fisher Scientific; Cat. # 462700250).

Perchloric Acid (70% HClO_4_) (Fujifilm Wako; Cat. # 162–00695).

Dansyl chloride (Fujifilm Wako; Cat. # 042-18254).

Acetone for HPLC (Acetone) (Fujifilm Wako; Cat. # 014-08681).

Proline (Sigma-Aldrich; Cat. # P0380-100 G).

Toluene (Fujifilm Wako; Cat. # 204–01866).

Acetonitrile (Fujifilm Wako; Cat. # 019-21691).

Sodium carbonate JIS special grade, ≥ 99.5% (Na_2_CO_3_) (Sigma-Aldrich; Cat. # 28-2170-5-500 G).

Sodium hydrogen carbonate JIS special grade, ≥ 99.5% (NaHCO_3_) (Sigma-Aldrich; Cat. # 28-1850-5-500 G).

Sodium chloride (NaCl) (Fujifilm Wako; Cat. # 195–01663).

Potassium chloride (KCl) (Fujifilm Wako; Cat. # 163–03545).

Sodium phosphate dibasic (Na_2_HPO_4_-12H_2_O) (Fujifilm Wako; Cat. # 196–02835).

Potassium phosphate monochloride (KH_2_PO_4_) (Fujifilm Wako; Cat. # 169–04245).

Ethylenediaminetetraacetic acid disodium salt dihydrate (EDTA) JIS special grade, ≥ 99.5% (Sigma-Aldrich; Cat. # 09-1420-5-500G-J).

Sucrose (Fujifilm Wako; Cat. # 196 − 00015).

(+/-)-Dithiothreitol (DTT) (Fujifilm Wako; Cat. # 045-08974).

Protease inhibitor Cocktail (Sigma-Aldrich; Cat. # P8340).

Formic Acid for LC/MS (Formic Acid) (Fujifilm Wako; Cat. # 063-04192).

### Equipment

Homogenizer Dounce (glass homogenizer) (357542, WHEATON; https://www.dwk.com/na/laboratory/brands/wheaton).

Bio clean bench (MCV-B91F, PHC Corporation; https://www.phchd.com/global/phc).

Water bath (SM-05R, TAITEC Corporation; https://e-taitec.com/).

CO_2_ Incubator (MC0-5AC, PHC Corporation; https://www.phchd.com/global/phc).

Micro Refrigerated Centrifuge (Model 3700, KUBOTA Corporation; https://www.kubota.com/).

Centrifugal Concentrator Spin Dryer Standard (centrifugal evaporator) (VC-96R, TAITEC Corporation; https://e-taitec.com/).

Cooling trap (non-freon tabletop type) (CA200, Yamato Scientific Co., Ltd.; https://www.yamato-scientific.com/).

Oil rotary vacuum pump (GLD-051, ULVAC KIKO. Inc.; https://ulvac-kiko.com/en/).

UHPLC system (Vanquish UHPLC System Binary Pump H, Thermo Fisher Scientific, https://www.thermofisher.com/jp/en/home/brands/thermo-scientific.html).

Mass spectrometer (Orbitrap Exploris 240 mass spectrometer, Thermo Fisher Scientific, https://www.thermofisher.com/jp/en/home/brands/thermo-scientific.html).

TraceFinder Ver. 5.1 (Thermo Fisher Scientific, https://www.thermofisher.com/jp/en/home/brands/thermo-scientific.html).

FreeStyle Ver. 1.8 (Thermo Fisher Scientific, https://www.thermofisher.com/jp/en/home/brands/thermo-scientific.html).

### Preparation of reagents

6 M HCl: add slowly 49.1 mL of HCl (37–38%) to 50.9 mL H_2_O.

Phosphate-buffered saline (PBS): dissolve 8.01 g NaCl, 0.2 g KCl, 3.63 g Na_2_HPO_4_-12H_2_O and 0.24 g KH_2_PO_4_ in 800 mL H_2_O. Adjusting the pH of the solution to 7.4 using 6 M HCl. Make up the final volume of 1 L with H_2_O.

25 mM Tris-HCl buffer (pH 7.5): dissolve 3.03 g Tris in 800 mL H_2_O. Adjusting the pH of the solution to 7.5 using 6 M HCl. Make up the final volume of 1 L with H_2_O.

Homogenization buffer: dissolve 5.125 g sucrose, 37.2 mg EDTA, 40 mg DTT and 100 µL of protease inhibitor in 100 mL of 25 mM Tris-HCl buffer (pH 7.5).

5 mM d_7_-ornithine: dissolve 1.27 mg d_7_-ornithine in 1.5 mL of H_2_O.

0.5 mM d_7_-ornithine: add 120 µL of 5 mM d_7_-ornithine to 1.080 mL of 25 mM Tris-HCl buffer (pH 7.5).

0.1 mM d_7_-ornithine: add 30 µL of 5 mM d_7_- ornithine to 1.470 mL of 25 mM Tris-HCl buffer (pH 7.5).

1 mM pyridoxal 5’-phosphate: dissolve 6.2 mg pyridoxal 5’-phosphate hydrate in 25 mL of 25 mM Tris-HCl buffer (pH 7.5).

1.5 N HClO_4_: add 20 mL of 70% HClO_4_ to 134.6 mL H_2_O.

10 mg/mL dansyl chloride solution: dissolve 65 mg dansyl chloride in 6.5 mL acetone.

Saturated sodium carbonate buffer: Add saturated Na_2_CO_3_ solution to saturated NaHCO_3_ solution until adjusting the pH of the solution to 9.0.

100 mg/mL proline: dissolve 120 mg proline in 1.2 mL water.

Acetonitrile-water mixture (1:1, v/v): mix 2.5 mL acetonitrile and 2.5 mL water.

0.2% formic acid-water mixture (v/v): mix 1 mL formic acid and 500 mL water.

0.2% formic acid-acetonitrile mixture (v/v): mix 1 mL formic acid and 500 mL acetonitrile.

### Polyamine standard solutions

LC–MS grade methanol-water mixture (1:1, v/v) is used to prepare the following solutions.

1. 50 mM putrescine: dissolve 8.05 mg putrescine dihydrochloride (MW = 161.1) in 1 mL methanol-water mixture.
  1 mM putrescine: mix 20 µL of 50 mM putrescine with 980 µL methanol-water mixture.
2. 50 mM spermidine: dissolve 12.73 mg spermidine trihydrochloride (MW = 254.6) in 1 mL methanol-water mixture.
  1 mM spermidine: mix 20 µL of 50 mM spermidine with 980 µL methanol-water mixture.
3. 50 mM spermine: dissolve 17.45 mg spermine tetrahydrochloride (MW = 348.2) in 1 mL methanol-water mixture.
  1 mM spermine: mix 20 µL of 50 mM spermine with 980 µL methanol-water mixture.

#### Mixed polyamine standards (100 µM for each polyamine)

Add the following to a microcentrifuge tube:

100 µL of 1 mM putrescine.

100 µL of 1 mM spermidine.

100 µL of 1 mM spermine.

700 µL methanol-water mixture.

Mixed 20 µM polyamine standards: Mix 200 µL of mixed 100 µM polyamine standards with 800 µL of methanol-water mixture.

Mixed 4 µM polyamine standards: Mix 200 µL of mixed 20 µM polyamine standards with 800 µL of methanol-water mixture.

Mixed 0.8 µM polyamine standards: Mix 200 µL of mixed 4 µM polyamine standards with 800 µL of methanol-water mixture.

Mixed 0.16 µM polyamine standards: Mix 200 µL of mixed 0.8 µM polyamine standards with 800 µL of methanol-water mixture.

Mixed 0.032 µM polyamine standards: Mix 200 µL of mixed 0.16 µM polyamine standards with 800 µL of methanol-water mixture.

#### Internal standard

LC–MS grade methanol-water mixture (1:1, v/v or 3:7, v/v) are used to prepare the following solutions.

1. 1 mg/mL d_8_-putrescine: dissolve 1 mg of d_8_-putrescine in 1:1 (v/v) methanol-water mixture.
2. 10 µM d_8_- putrescine: mix 40 µL of 1 mg/mL d_8_-putrescine in 4.132 mL of 3:7 (v/v) methanol-water mixture.
3. 1.5 N HClO_4_ and IS mix: mix 19 mL of 1.5 N HClO_4_ and 1 mL of 10 µM d_8_-putrescine.

### Chromatographic equipment

The LC–MS analysis is performed using a UHPLC system (Vanquish UHPLC System Binary Pump H) equipped with a high-resolution MS (Orbitrap Exploris 240 mass spectrometer). The dansyl-derivatized polyamines are separated with column (Accucore™ Vanquish™ C18+ (100 × 2.1 mm, 1.5 μm); P/N 27101–102130; Thermo Fisher Scientific) at 40 °C. The mobile phase (MP) was MP-A (water containing 0.2% formic acid) and MP-B (acetonitrile containing 0.2% formic acid), and flow rate was set as 0.4 mL/min. The UHPLC is run at a gradient mode (Table 1). The mass spectrometer was operated in positive mode using an ESI source using scan range of 150-2,000 *m*/*z*. Both LC–MS and LC–MS/MS data were acquired; however, quantitative analysis in this study was performed using LC–MS data. The mass spectral data were analyzed by TraceFinder and FreeStyle.

**Table 1:** LC–MS gradient conditions used for the analysis of dansyl-derivatized polyamines

Solvent (%)	Time (min)					
	0	1	8	11	11.01	12
MP-A	50	50	0	0	50	50
MP-B	50	50	100	100	50	50

The total run time is 12 min. The mobile phase (MP) consisted of MP-A (water containing 0.2% formic acid) and MP-B (acetonitrile containing 0.2% formic acid), with a flow rate of 0.4 mL/min

Quantification of d_7_-putrescine was performed using an internal standard-based external calibration method. Calibration curves were constructed using known concentrations of unlabeled putrescine and a fixed amount of d_8_-putrescine as the internal standard. The peak area ratio of d_7_-putrescine (or unlabeled putrescine) to d_8_-putrescine was used for quantification.

## Procedures

**Fig. 2:**
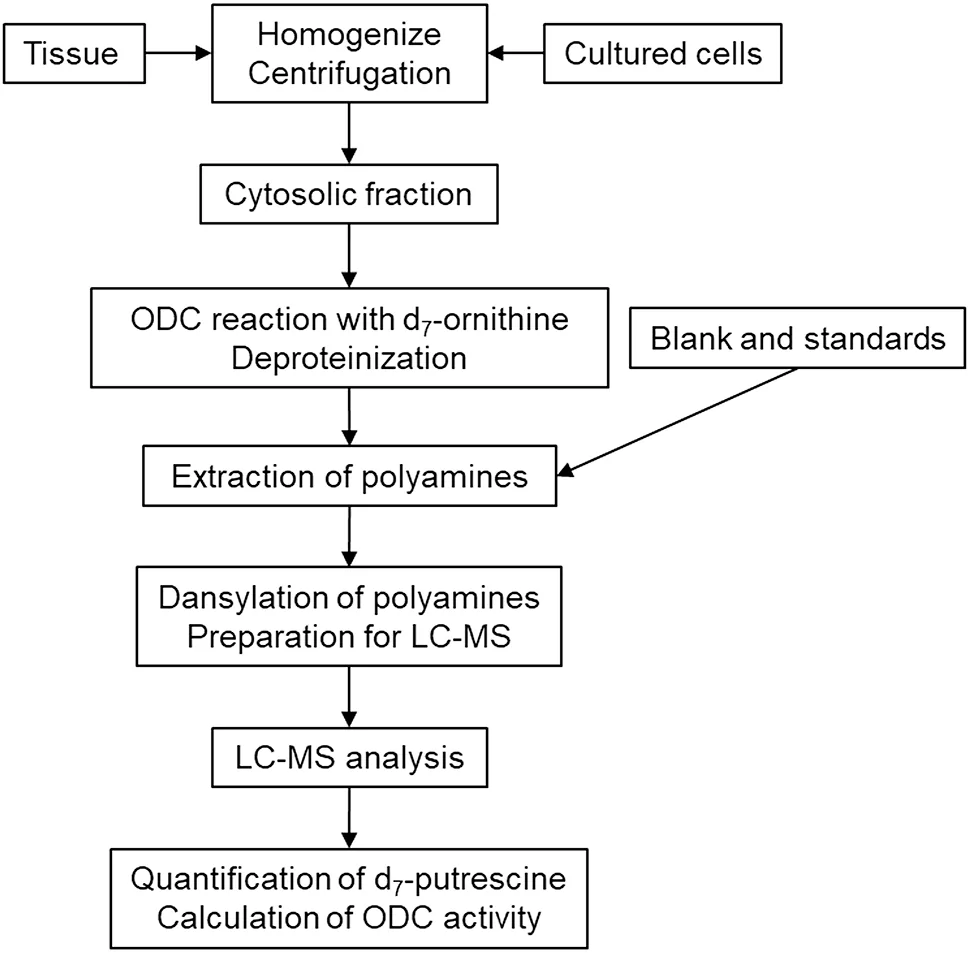
Workflow diagram of the procedures for ODC reaction and LC–MS-based analysis. Cytosolic fraction was prepared from biological tissues or cells by acid extraction. The samples were incubated with d_7_-ornithine, and the produced d_7_-putrescine was quantified by LC–MS following dansyl derivatization to calculate ODC activity

The overall workflow of the ODC reaction and the LC–MS–based analysis is summarized in Fig. 2.

### Sample preparations for enzyme reaction

#### Preparation of tissues

1. Add approximately 0.5 g of each fresh tissue to 2 mL of ice-cold homogenization buffer in glass homogenizer.
2. Grind fresh tissue.
3. Transfer the homogenate to 2 mL Eppendorf tube and centrifuge at 600 × g for 10 min at 4 °C.
4. Transfer the supernatant fluid and further centrifuge at 10,000 × g for 10 min at 4 °C.
5. Transfer the second supernatant (cytosolic fraction) to new 2 mL Eppendorf tube.

The protein concentration of cytosolic fraction is approximately 30 mg/mL.

Previous studies have reported that the ODC concentration in rat liver is 0.18 ± 0.02 ng/mg protein or 0.35 ± 0.20 ng/mg protein (Kanamoto et al. 1991; Tomiya et al. 1991).

#### Preparation of cultured cells

1. Approximately 2.0 × 10^6^ cells are washed twice with PBS.
2. Scrape into 1 mL homogenization buffer and disrupt by sonication.
3. Transfer the cell solution to 2 mL Eppendorf tube and centrifuge at 600 × g for 10 min at 4 °C.
4. Transfer the supernatant fluid and further centrifuge at 10,000 × g for 10 min at 4 °C.
5. Transfer the second supernatant (cytosolic fraction) to new 2 mL Eppendorf tube.

The protein concentration of cytosolic fraction is approximately 2 mg/mL.

**Note:** The homogenization buffer and glass homogenizer are kept cooled on ice. The volume of homogenization buffer should be adjusted according to the number of cells or the weight of the tissue samples.

### Ornithine Decarboxylase (ODC) reaction

#### Test sample

1. Add 100 µL of 1 mM pyridoxal 5’-phosphate to the Eppendorf tube and vortex.
2. Add 100 µL of 0.1 mM or 0.5 mM d_7_-ornithine to this Eppendorf tube and vortex.
3. Add 100–300 µL of the protein solution (cytosolic fraction) to this Eppendorf tube and adjust the total volume to 300 µL with 25 mM Tris‑HCl buffer (pH 7.5). Mix gently.
4. Incubate at 37 °C in water bath.
5. Add 500 µL of 1.5 N HClO_4_ and IS mix and vortex to terminate enzyme reaction.
6. Keep on ice for 10 min.
7. Centrifuge the tube at 10,000 × g for 5 min at 4 °C.
8. Transfer the supernatant fluid to a new 1.5 mL Eppendorf tube.

We determined the reaction pH and concentration of pyridoxal-5’-phosphate based on previously established protocols for measuring ODC activity using radioisotope-labeled substrates (Furukawa et al. 2021; Ducros et al. 2009).

#### Negative control (the deproteinized sample before enzyme reaction)

1. Add 100 µL of 1 mM pyridoxal 5’-phosphate to the Eppendorf tube and vortex.
2. Add 100 µL of 0.1 mM or 0.5 mM d_7_-ornithine to this Eppendorf tube and vortex.
3. Add 100–300 µL of the protein solution (cytosolic fraction) to this Eppendorf tube and adjust the total volume to 300 µL with 25 mM Tris‑HCl buffer (pH 7.5). Mix gently.
4. Add 500 µL of 1.5 N HClO_4_ and IS mix and vortex to deproteinized ODC before enzyme reaction.
5. Incubate at 37 °C in water bath.
6. Keep on ice for 10 min.
7. Centrifuge the tube at 10,000 × g for 5 min at 4 °C.
8. Transfer the supernatant fluid to a new 1.5 mL Eppendorf tube.

#### External standards and blank preparation

1. Add 50 µL of 1.5 N HClO_4_ and IS mix to 50 µL of Mixed polyamine standards (20, 4, 0.8, 0.16, 0.032 µM) and vortex (external standards). Add 50 µL of 1.5 N HClO_4_ and IS mix to 50 μL of H_2_O and vortex (blank).
2. Keep on ice for 10 min.
3. Centrifuge the tube at 10,000 × g for 5 min at 4 °C.
4. Transfer the supernatant fluid to a new 1.5 mL Eppendorf tube.

### Dansylation procedure

The polyamines are derivatized with dansyl chloride with minor modification (Ducros et al. 2009).

1. Transfer 50 µL of the supernatant fluid (reaction samples, external standards or blank) to a new 1.5 mL Eppendorf tube.
2. Add 150 µL of a saturated sodium carbonate buffer (pH 9.0) and vortex.
3. Add 300 µL of 10 mg/mL dansyl chloride solution and vortex, and incubate at 70 °C for 15 min.
4. Add 50 µL of 100 mg/mL proline and vortex, and incubate at 25 °C for 30 min.
5. Add 500 µL of toluene and vortex.
6. Centrifuge the tube at 10,000 × g for 5 min at 4 °C.
7. Transfer 500 µL of the organic phase (upper fraction) to a new 1.5 mL Eppendorf tube.
8. Leave the cap open and evaporate to dryness using centrifugal evaporator equipped with a cooling tarp and an oil rotary vacuum pump.
9. Re-dissolve the residue in 200 µL of acetonitrile-water mixture (1:1, v/v).
10. Transfer 50 µL to LC–MS vials and analysis.

**Note:** Dansyl derivatization proceeds only under basic conditions. Therefore, the pH of the reaction mixture should be checked prior to incubation. If the pH is not sufficient basic, add sodium carbonate buffer to adjust the pH accordingly.

### LC–MS chromatograms

**Fig. 3:**
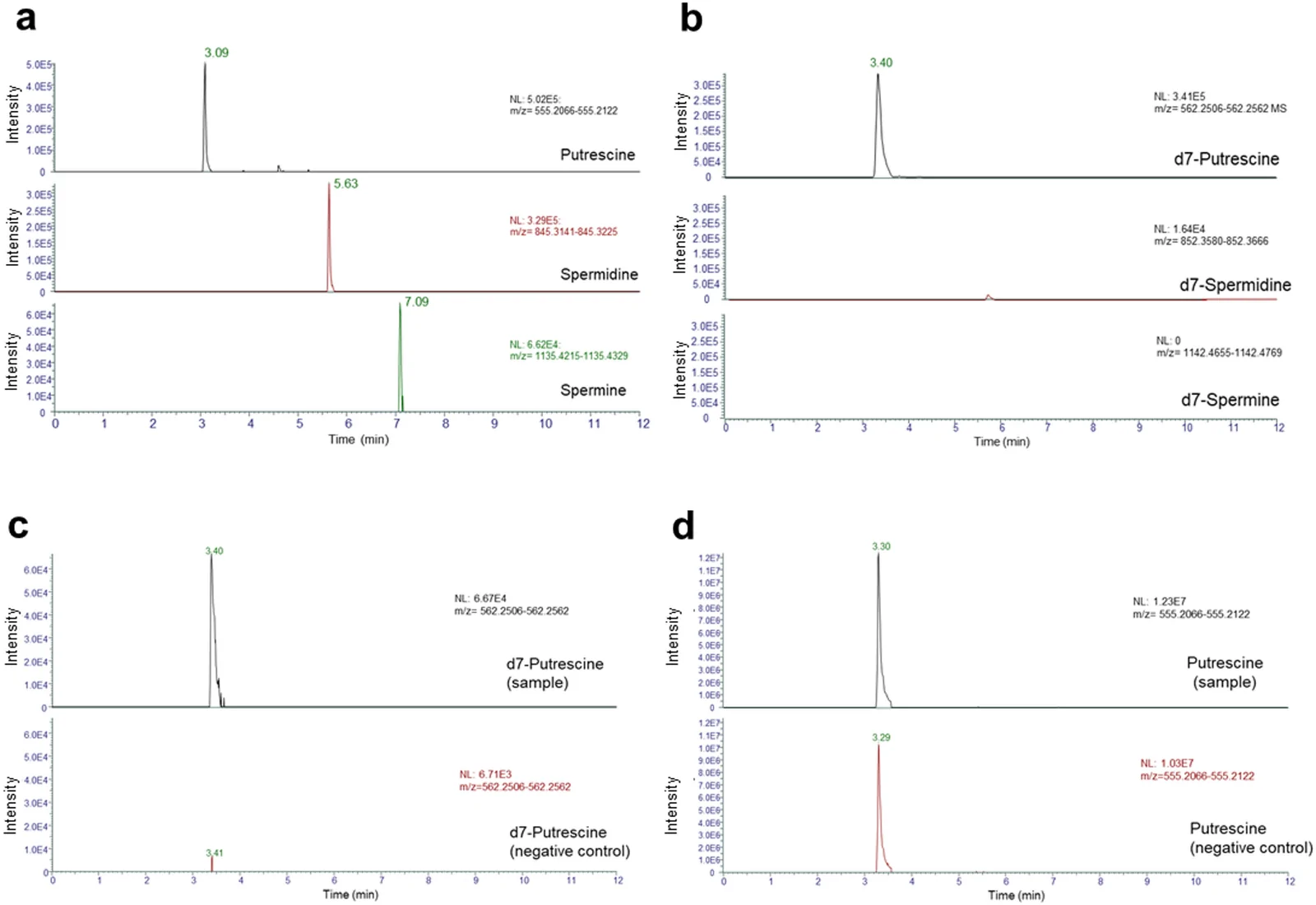
Chromatograms for dansyl-derivatized polyamines using LC–MS. Polyamines (putrescine, spermidine and spermine) were derivatized with dansyl chloride and analyzed by LC–MS. **a** Mass chromatograms of derivatized putrescine, spermidine and spermine in a calibration standard. **b** Mass chromatograms of d_7_-labeled putrescine, spermidine and spermine in a biological sample. **c** Peak corresponding to d_7_-putrescine produced from d_7_-ornithine used as the reaction substrate, compared with its negative control. **d** Peak corresponding to non-labeled putrescine produced from non-labeled ornithine used as the reaction substrate, compared with its negative control. The vertical axis represents peak intensity, and the horizontal axis represents retention time. Polyamines were identified based on their reference *m*/*z* range (putrescine: 555.2066-555.2122, spermidine: 845.3141-845.3225, spermine: 1135.4215-1135.4329, d_7_-putrescine: 562.2506-562.2562, d_7_-spermidine: 852.3580-852.3666, d_7_-spermine: 1142.4655-1142.4769)

As shown in Fig. 3a, the dansyl-derivatized polyamine standards were well separated and exhibited sharp chromatographic peaks. Under the established LC‑MS conditions, no significant interfering peaks were observed at the retention time corresponding to putrescine, indicating adequate specificity for putrescine quantification. In our preliminary experiment, underivatized polyamines in calibration standard were detectable by LC–MS (Supplementary Fig. 1a); however, our protocol failed to achieve stable detection in biological samples, particularly those derived from animal tissues (Supplementary Fig. 1b). Therefore, we concluded that the derivatization step is essential for reliable quantification. Several derivatization methods for polyamine detection have been reported, including isobutyl chloroformate (Magnes et al. 2014), benzoyl chloride (Liu et al. 2012), N-(9-Fluorenylmethoxycarbonyloxy) succinimide (Xiong and Zhai 2016), and 4-(N, N-dimethylaminosulfonyl)7-fluoro-2,1,3-benzoxadiazole (DBD-F) (Tsutsui et al. 2013). In addition to dansyl chloride, we also evaluated isobutyl chloroformate as an alternative derivatization reagent. As shown in Supplementary Fig. 1c and 1d, isobutyl chloroformate-derivatized polyamines were readily detected in both calibration standards and biological samples, indicating that multiple derivatization strategies are applicable for LC–MS-based polyamine quantification. Among these methods, derivatization with dansyl chloride is simple, cost-effective, and widely used in the literatures. Therefore, dansyl chloride was selected as the derivatization reagent for the present study.

### Analytical validation of putrescine quantification by LC–MS

The analytical performance of the method was evaluated in terms of method detection limit (MDL), limit of quantification (LOQ), interday precision, recovery, and matrix effects.

### Method detection limit (MDL), limit of quantification (LOQ), and interday precision

The MDL and LOQ were determined using kidney and plasma of rats. Seven replicate samples with putrescine concentrations near the estimated detection limit were analyzed, and the standard deviation (SD) was calculated. The MDL was defined as 3.14 × SD and the LOQ was calculated to be 10 × SD, based on the t-value for six degrees of freedom and a 99% confidence level.

As summarized in Table 2, the MDL of this assay were 7.84 pmol/mg tissue (kidney) and 0.20 nmol/ml (plasma), and the LOQ of this assay were 24.98 pmol/mg tissue (kidney) and 0.65 nmol/ml (plasma).

**Table 2:** Method detection limit (MDL), limit of quantification (LOQ), and interday precision for detecting putrescine in the biological matrices

	MDL	LOQ	Interday precision
Rat kidney	7.84 pmol/mg tissue	24.98 pmol/mg tissue	7.10%
Rat plasma	0.20 nmol/ml	0.65 nmol/ml	2.80%

MDL and LOQ were determined using rat kidney and plasma samples. Putrescine levels in seven replicate samples near the detection limit were measured, and the standard deviation was used for calculation. MDL and LOQ values are normalized by tissue weight (mg) or plasma volume (ml). Interday precision was evaluated by measuring putrescine level in rat kidney and plasma over seven consecutive days and express as relative standard deviation (%RSD)

These values indicate sufficient analytical sensitivity to detect low‑abundance putrescine in biological samples.

Interday precision was evaluated by analyzing the same kidney and plasma samples on seven consecutive days. Precision was expressed as the relative standard deviation (RSD). As shown in Table 2, the RSD values were within acceptable ranges, demonstrating method reproducibility for routine analysis.

### Matrix effects and recovery

Matrix effects were assessed by comparing the putrescine concentration in matrix samples with those prepared in solvent according to equation.

$$\:\mathrm{M}\mathrm{a}\mathrm{t}\mathrm{r}\mathrm{i}\mathrm{x}\:\mathrm{e}\mathrm{f}\mathrm{f}\mathrm{e}\mathrm{c}\mathrm{t}\mathrm{s}\:\left(\mathrm{\%}\right)=100\:\times\:\frac{\mathrm{p}\mathrm{u}\mathrm{t}\mathrm{r}\mathrm{e}\mathrm{s}\mathrm{c}\mathrm{i}\mathrm{n}\mathrm{e}\:\mathrm{c}\mathrm{o}\mathrm{n}\mathrm{c}\mathrm{e}\mathrm{n}\mathrm{t}\mathrm{r}\mathrm{a}\mathrm{t}\mathrm{i}\mathrm{o}\mathrm{n}\:\left(\mathrm{m}\mathrm{a}\mathrm{t}\mathrm{r}\mathrm{i}\mathrm{x}\right)}{\mathrm{p}\mathrm{u}\mathrm{t}\mathrm{r}\mathrm{e}\mathrm{s}\mathrm{c}\mathrm{i}\mathrm{n}\mathrm{e}\:\mathrm{c}\mathrm{o}\mathrm{n}\mathrm{c}\mathrm{e}\mathrm{n}\mathrm{t}\mathrm{r}\mathrm{a}\mathrm{t}\mathrm{i}\mathrm{o}\mathrm{n}\:\left(\mathrm{s}\mathrm{o}\mathrm{l}\mathrm{v}\mathrm{e}\mathrm{n}\mathrm{t}\right)}$$

As shown in Table 3, matrix effect for rat kidney ranges 115.88 to 118.69%, indicating moderate signal enhancement but acceptable ionization behavior under the present conditions.

**Table 3:** Matrix effects and recovery for detecting putrescine in the biological matrices (rat kidney)

	Fortified level of putrescine (µM)	Matrix effects (%)	Recovery (%)
Rat kidney	0.8	115.9	91.4
	4	118.7	94.4
	20	118.0	90.5

Matrix effects were evaluated by comparing putrescine concentrations in solvent and biological matrix. Recovery was assessed by comparing concentrations obtained from samples spiked before and after deproteinization

Recovery was evaluated by comparing the putrescine concentration in the matrix samples fortified before and after deproteinizing step using the following equation.

$$\:\mathrm{R}\mathrm{e}\mathrm{c}\mathrm{o}\mathrm{v}\mathrm{e}\mathrm{r}\mathrm{y}\:\left(\mathrm{\%}\right)=100\:\times\:\frac{\mathrm{p}\mathrm{u}\mathrm{t}\mathrm{r}\mathrm{e}\mathrm{s}\mathrm{c}\mathrm{i}\mathrm{n}\mathrm{e}\:\mathrm{c}\mathrm{o}\mathrm{n}\mathrm{c}\mathrm{e}\mathrm{n}\mathrm{t}\mathrm{r}\mathrm{a}\mathrm{t}\mathrm{i}\mathrm{o}\mathrm{n}\:\mathrm{b}\mathrm{e}\mathrm{f}\mathrm{o}\mathrm{r}\mathrm{e}\:\mathrm{d}\mathrm{e}\mathrm{p}\mathrm{r}\mathrm{o}\mathrm{t}\mathrm{e}\mathrm{i}\mathrm{n}\mathrm{i}\mathrm{z}\mathrm{i}\mathrm{n}\mathrm{g}}{\mathrm{p}\mathrm{u}\mathrm{t}\mathrm{r}\mathrm{e}\mathrm{s}\mathrm{c}\mathrm{i}\mathrm{n}\mathrm{e}\:\mathrm{c}\mathrm{o}\mathrm{n}\mathrm{c}\mathrm{e}\mathrm{n}\mathrm{t}\mathrm{r}\mathrm{a}\mathrm{t}\mathrm{i}\mathrm{o}\mathrm{n}\:\mathrm{a}\mathrm{f}\mathrm{t}\mathrm{e}\mathrm{r}\:\mathrm{d}\mathrm{e}\mathrm{p}\mathrm{r}\mathrm{o}\mathrm{t}\mathrm{e}\mathrm{i}\mathrm{n}\mathrm{i}\mathrm{z}\mathrm{i}\mathrm{n}\mathrm{g}}$$

Recovery ranged from 90.46 to 94.40%, indicating consistent and efficient analyte recovery during sample preparation.

### Detection of d_7_-putrescine after ODC enzyme reactions

Figure 3b shows the chromatograms of d_7_-putrescine, d_7_-spermidine, and d_7_-spermine from ODC reaction mixtures prepared using rat kidney cytosolic fraction. A strong peak corresponding to d_7_-putrescine was detected, whereas negligible or no peaks were observed for d_7_-spermidine and d_7_-spermine. Because the conversion of putrescine to spermidine and spermidine to spermine requires decarboxylated S-adenosylmethionine (Wu 2013), these downstream reactions did not proceed under our enzymological conditions. Taken together, these results indicate that ODC activity can be calculated based on the quantitative level of synthesized d_7_-putrescine.

Note that, in addition to ODC reactions, ornithine aminotransferase and ornithine transcarbamylase also act on ornithine-catalyzed enzymes in mitochondria (Jackson et al. 1986; Ginguay et al. 2017). However, because cytosolic fractions were used in this assay, these mitochondrial enzymes would not affect the measurement of ODC.

Since stable isotopes occur naturally, a minor peak corresponding to d_7_-putrescine (*m*/*z* = 562.2) was detected even in the negative control (Fig. 3c bottom). However, this peak was markedly smaller than that in the ODC reaction sample (Fig. 3c top). In contrast, when non-labeled ornithine was used as a substrate for ODC enzyme reaction, non-labeled putrescine (*m*/*z* = 555.2) was detected in the reacted sample (Fig. 3d top), while its peak intensity was comparable to that of the negative control (Fig. 3d bottom). Given that ODC activity is relatively low compared with other enzymes (e.g., POX and ARG) (Furukawa et al. 2021), and that endogenous polyamines are abundant in tissues and cultured cells, newly synthesized putrescine cannot be reliably distinguished from background signal when non-labeled ornithine is used. Therefore, the use of stable isotope-labeled ornithine represents the optimal approach for accurate quantification of ODC activity.

### Determination of optimal condition for ODC reaction

**Fig. 4:**
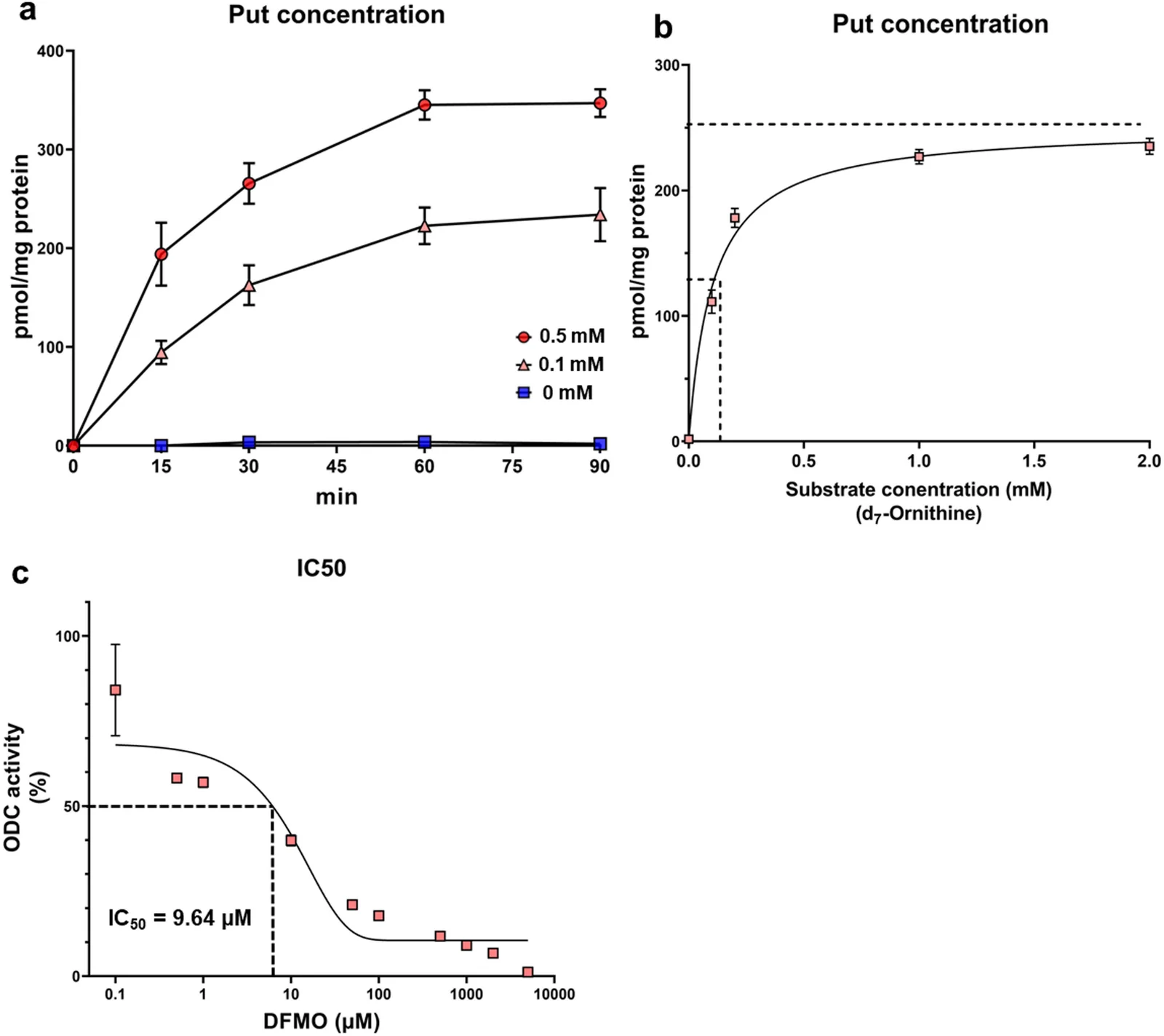
Optimization of reaction conditions and validation of the ODC assay. ODC reactions were carried out using rat kidneys with d_7_-ornithine, and the produced d_7_-putrescine (Put) was quantified by LC–MS following the dansyl derivatization. **a** Time-course analysis of d_7_-putrescine (0, 15, 30, 60, and 90 min) under different substrate concentration (0 mM, 0.1 mM, and 0.5 mM of d_7_-ornithine). **b** Dependence of d_7_‑putrescine production on d_7_‑ornithine concentration, measured after 60 min of incubation. **c** ODC activities in AML12 cells after 48 h treatment with increasing concentrations of the ODC inhibitor difluoromethylornithine (DFMO). IC_50_ value was calculated by nonlinear regression analysis using a four-parameter logistic model. Data are presented as mean ± standard error (SE), with *n* = 3

Accurate evaluation of ODC activity requires appropriate control of both substrate concentration and reaction time. We therefore monitored the production of d_7_-putrescine using 0, 0.1, or 0.5 mM of d_7_-ornithine as a substrate (Fig. 4a). Samples incubated with 0.5 mM d_7_-ornithine exhibited higher levels of d_7_-putrescine compared with those treated with 0.1 mM d_7_-ornithine at all-time points, whereas negligible d_7_-putrescine was detected in the absence of substrate (0 mM d_7_-ornithine). Regarding reaction time, the amount of d_7_-putrescine increased linearly from 0 to 60 min at 0.1 and 0.5 mM d_7_-ornithine, after which the levels plateaued between 60 and 90 min.

Using the linear reaction endpoint at 60 min, the dependence of d7-putrescine production on d7-ornithine concentration was further examined over a wider substrate range, and the apparent Michaelis constant (Km) was estimated. As shown in Fig. 4b, the apparent Km value was estimated to be 0.104 mM. Based on this result, a substrate concentration of 0.5 mM d_7_‑ornithine was considered sufficient to ensure efficient progression of the ODC reaction. Taken together, incubation with 0.5 mM d7‑ornithine for 60 min provides a robust and clearly quantifiable measurement of ODC activity.

- 

### Quantification of ODC reaction

To quantify the ODC activity, the below calculation was performed.

$$\begin{aligned} & {\mathrm{ODC~activity~}}\left( {{\mathrm{pmol}}/{\mathrm{mg~protein}}/{\mathrm{h}}} \right){\mathrm{~}} \\ & \quad = {\mathrm{~}}\frac{{d_{7}{\mathrm{~putrescine~of~test~sample~}}\left( {{\mathrm{pmol}}} \right) - d_{7}{\mathrm{~putrescine~of~negative~control~}}\left( {{\mathrm{pmol}}} \right){\mathrm{~}}}}{{{\mathrm{Protein~concentration~}}\left( {{\mathrm{mg}}/{\mathrm{mL}}} \right){\mathrm{~}} \times {\mathrm{~Sample~amount~}}\left( {{\mathrm{mL}}} \right){\mathrm{~}} \times {\mathrm{~Reaction~time~}}\left( {\mathrm{h}} \right)}} \\ \end{aligned} $$

We confirmed that the linear range of the calibration curve for putrescine was 0.032–20 µM (R^2^ = 0.99), indicating that the lower limit of quantification for d_7_-putrescine concentration was 0.032 µM. Based on this value and the assay conditions (total reaction volume, protein amount and incubation time), the lower limit of quantifiable ODC activity in the present assay was estimated to be approximately 5 pmol/mg protein/h.

### Precision and practicality of this ODC assay

Having established the optimal assay conditions, we next examined whether the method could be extended and validated in cultured cells (mouse AML12 hepatocytes). Despite the limited protein yield from cultured cells (~ 0.6 mg), d_7_-putrescine was readily detectable, allowing accurate quantification of ODC activity (Fig. 4c). This result indicates that our ODC assay is applicable even when only limited amounts of protein are available, such as cultured cells. We further examined whether this assay accurately responds to conditions that suppress ODC activity. As we expected, ODC activity of AML12 cells was inhibited by DFMO (ODC inhibitor) treatment in a dose-dependent manner (Fig. 4c). Based on nonlinear regression analysis, the IC₅₀ value was estimated to be 9.64 µM under the present assay conditions. This result indicates that our ODC assay accurately reflects the pharmacological inhibition.

Finally, to confirm broader applicability to biological samples, ODC activities were quantified using the established assay in the liver, kidney, jejunum and spleen obtained from rats, as well as in the kidney obtained from 0- and 14- day-old chickens (Table 4). The ODC activity values obtained from broiler chicken kidney in this study were in agreement with those reported in our previous study using a radiolabeled method (Furukawa et al. 2021), despite the differences in analytical approaches.

**Table 4:** Quantification of ODC activity in animal tissue samples using the established LC–MS assay

	ODC activity (pmol/mg protein/h)
Rat liver ^a^	85.2 ± 15.1
Rat kidney ^a^	349.1 ± 95.7
Rat jejunum ^a^	19.6 ± 3.7
Rat spleen ^a^	62.5 ± 25.0
Chicken (Day 0) kidney ^b^	46.0 ± 16.3
Chicken (Day 14) kidney ^b^	199.7 ± 36.7

Values are presented as mean ± SEM, *n* = 3

^a^Tissue samples were obtained from 6-week-old male Wistar rats

^b^Tissue samples were obtained from male broiler chickens (ROSS strain)

## Supplementary Information

Below is the link to the electronic supplementary material.


Supplementary Material 1


## Data Availability

No datasets were generated or analysed during the current study.
