# Supplementary material for: LC–MS assay for quantifying ornithine decarboxylase activity in the biological matrix and cultured cells using stable isotope‑labeled ornithine
Source: Amino Acids. 2026 Jun 21;58:46. doi: 10.1007/s00726-026-03539-9 (PMC13534377; doi:10.1007/s00726-026-03539-9)
Supplement: Supplementary file 1 [file 726_2026_3539_MOESM1_ESM.docx]

**Supplemental Fig. 1** Detection of underivatized polyamines and polyamines derivatized with isobutyl chloroformate.

Polyamines (putrescine, spermidine and spermine) in calibration standards and plasma samples from 7-day-old broiler chickens (Ross strain) were analyzed by LC-MS with or without derivatization. (**a**) Mass chromatograms of underivatized putrescine, spermidine and spermine in a calibration standard. (**b**) Mass chromatograms of underivatized putrescine, spermidine and spermine in a plasma sample. (**c**) Mass chromatograms of polyamines derivatized with isobutyl chloroformate (IBCF) in a calibration standard. (**d**) Mass chromatograms of polyamines derivatized with IBCF in a plasma sample. The vertical axis represents peak intensity, and the horizontal axis represents retention time. Polyamines were identified based on their reference m/z range (put: 89.1069-89.1077, spd: 146.1645-146.1659, spn: 203.2220-203.2240, IBCF-putrescine: 289.2108-289.2136, IBCF-spermidine: 446.3203-446.3247, IBCF-spermine: 603.4297-603.4357).
